# Environmental enrichment as a mitochondria-targeting systems strategy across neurodegenerative diseases and retinal dystrophies

**DOI:** 10.3389/fnins.2026.1744873

**Published:** 2026-01-20

**Authors:** Dario Rusciano, Caterina Gagliano, Alessandro Avitabile, José Fernando Maya-Vetencourt

**Affiliations:** 1 Retired, Catania, Italy; 2 Faculty of Medicine and Surgery, University of Enna “Kore”, Enna, Italy; 3 Mediterranean Foundation “G.B. Morgagni”, Catania, Italy; 4 Neurovisual Science Technology (NEST), Catania, Italy; 5 Department of Biology, Physiology Institute, University of Pisa, Pisa, Italy

**Keywords:** environmental enrichment, mitochondria, neurodegeneration, neuronal plasticity, oxidative stress, retinal dystrophy, visual system

## Abstract

Mitochondrial dysfunction is a central contributor to neurodegenerative disorders affecting both the central nervous system and the retina, where impaired energy metabolism, oxidative stress, and defective cellular resilience converge to drive progressive neuronal loss. Environmental enrichment (EE), a multimodal non-pharmacological paradigm, has emerged as a powerful modulator of brain and retinal plasticity in preclinical models, promoting adaptive responses that support mitochondrial function and neurotrophic signaling. This review synthesizes evidence indicating that EE influences mitochondrial quality control, redox homeostasis, synaptic resilience, and neuroimmune balance across a range of experimental models of neurodegeneration and retinal dystrophy. While these effects converge on shared downstream pathways, important disease-, cell-type-, and context-specific differences exist, and mechanistic generalization across systems requires caution. Human studies remain limited, heterogeneous, and often focused on functional outcomes rather than direct biological endpoints, resulting in modest and variable effect sizes. Rather than proposing EE as a stand-alone therapy, we frame it as a system-level, disease-modifying context that may enhance endogenous protective capacity and potentially complement pharmacological, genetic, or rehabilitative interventions, pending disease-specific validation. Forward-looking perspectives, including digitally mediated and AI-supported EE-inspired approaches, are discussed as conceptual strategies whose biological relevance will depend on future studies integrating functional outcomes with validated molecular and metabolic biomarkers. Together, the available evidence positions EE as a biologically grounded, non-invasive framework for promoting neuro- and retino-protective resilience, while underscoring the need for rigorously designed translational and clinical studies to define its therapeutic boundaries and mechanisms of action.

## Introduction

Neurodegenerative and retinal degenerative diseases, while clinically and anatomically distinct, share partially overlapping pathological features, including mitochondrial dysfunction and oxidative stress, whose relative contribution varies across disease contexts and cell types. Alzheimer’s disease (AD), Parkinson’s disease (PD), Huntington’s disease (HD), and amyotrophic lateral sclerosis (ALS) primarily target the central nervous system (CNS), whereas retinitis pigmentosa (RP), age-related macular degeneration (AMD), glaucoma, and diabetic retinopathy (DR) affect the retina. Despite such differences, these disorders share a central vulnerability: impaired mitochondrial function and redox imbalance, which compromise energy metabolism, synaptic resilience, and cellular survival in both neurons and photoreceptors, driving progressive degeneration (Yang et al., 2024; Duarte, 2021).

In the CNS, mitochondrial impairment and reactive oxygen species (ROS) accumulation accelerate protein misfolding, synaptic dysfunction, and neuronal death. Similarly, the retina—particularly its photoreceptors—ranks among the most metabolically demanding tissues in the body, rendering it intrinsically sensitive to mitochondrial perturbations. The retinal pigment epithelium (RPE) plays a critical supportive role by regulating mitochondrial function, redox balance, and metabolic coupling essential for photoreceptor homeostasis. Dysfunction across these interdependent cellular compartments disrupts ATP production, increases ROS, and activates apoptotic pathways, contributing to progressive degeneration. Conventional pharmacologic, gene-based, or surgical therapies remain largely symptomatic or mutation-specific and are often limited to advanced disease stages, underscoring the need for broader, non-invasive strategies that address shared upstream mechanisms (Kharat et al., 2024).

Environmental enrichment (EE), which combines enhanced physical activity, cognitive stimulation, and social interaction, emerges as a compelling non-pharmacological and non-invasive strategy (Sale et al., 2014). Therefore, EE is inherently multimodal, integrating physical activity, cognitive challenge, social interaction, and sensory stimulation; as such, its biological effects often reflect emergent interactions rather than the sum of isolated components. In experimental models of neurodegeneration, EE improves mitochondrial dynamics, reduces oxidative stress, and enhances neurotrophic signaling, with disease- and tissue-specific manifestations. In Alzheimer’s models, EE delays cognitive decline, reduces amyloid-beta pathology, and mitigates oxidative stress by boosting antioxidant defenses and suppressing pro-apoptotic and pro-inflammatory mediators (Berardi et al., 2007; Nithianantharajah and Hannan, 2006; Herring et al., 2010; Liew et al., 2022). EE matches the protective effects of physical exercise and outperforms social enrichment alone in preserving memory, preventing lipid peroxidation, and restoring hippocampal synaptic function in models of chronic cerebral hypoperfusion (Prado Lima et al., 2018; Xu et al., 2023). In Parkinsonian models, EE prevents dopaminergic neuronal loss (Faherty et al., 2025); in Huntington’s disease models, it rescues protein deficits, preserves brain-derived neurotrophic factor (BDNF) levels, and maintains motor and cognitive function (Spires et al., 2004). Remarkably, EE also confers retinal protection: in retinitis pigmentosa models, it prolongs photoreceptor survival and preserves visual function through ciliary-neurotrophic-factor (CNTF) upregulation and mammalian-Target-of-Rapamycin (mTOR) pathway activation (Barone et al., 2012).

Mechanistically, EE enhances mitochondrial function, promotes antioxidant defenses, stimulates autophagic clearance of damaged organelles, and supports neurotrophic signaling, while reducing oxidative and apoptotic stress. By targeting shared pathological mechanisms, EE offers a unifying therapeutic strategy capable of bridging neurodegeneration and retinal degeneration. This review synthesizes evidence supporting EE as a mitochondrial-centric intervention, highlighting its impact on mitochondrial dynamics, oxidative stress, neurotrophic signaling, inflammation, and synaptic integrity across CNS and retinal models, while discussing translational potential and limitations. However, although EE consistently modulates molecular pathways linked to mitochondrial biogenesis, mitophagy, and antioxidant defenses, most evidence remains indirect and associative rather than causally demonstrative. EE should therefore not be interpreted as a mitochondrial therapy in the pharmacological sense, but as a systems-level intervention that recruits mitochondrial pathways as part of a broader adaptive response.

Moreover, while this review integrates evidence from CNS neurodegenerative disorders and retinal dystrophies, these conditions are not assumed to be biologically equivalent. Differences in cellular composition, metabolic demand, circuit architecture, and disease tempo critically shape how EE is implemented and interpreted across systems. The retina, in particular, is a highly specialized neural tissue characterized by extreme mitochondrial dependence, continuous sensory activation, and unique glial–neuronal interactions. Accordingly, EE-related mechanisms discussed herein should be understood as convergent at the level of core stress-response and plasticity pathways, while remaining disease- and cell-type specific in their upstream triggers and downstream functional outcomes.

Because several signaling pathways—including Brain-Derived Neurotrophic Factor (BDNF), the AMP-activated protein kinase–Sirtuin 1–Peroxisome proliferator-activated receptor gamma coactivator 1-alpha axis (AMPK–SIRT1–PGC-1α), and redox-regulatory networks—act as integrative hubs across plasticity, metabolism, and neurodegeneration, they are discussed in multiple sections from distinct conceptual perspectives. Where possible, repetition has been minimized while retaining key elements necessary for conceptual continuity.

## Mitochondrial dysfunction in neurodegeneration

### Mitochondrial biology in the CNS

Mitochondria are highly dynamic organelles essential for neuronal survival, coordinating ATP production, calcium buffering, and redox homeostasis. Their plasticity relies on continuous cycles of fission, fusion, biogenesis, and mitophagy, collectively known as mitochondrial dynamics (Giacomello et al., 2020). Disruption of these processes, particularly mitophagy, leads to oxidative stress accumulation and bioenergetic failure, key drivers of neurodegenerative pathology (Wang et al., 2023). Mitochondrial fusion, mediated by the outer membrane GTPases Mitofusin 1 and Mitofusin 2 (MFN1/2) and the inner membrane dynamin-related protein Optic Atrophy 1 (OPA1), enables the exchange of mitochondrial contents to compensate for local damage. Conversely, mitochondrial fission, regulated by the cytosolic GTPase Dynamin-Related Protein 1 (DRP1) and its mitochondrial receptor Mitochondrial Fission Factor (MFF), isolates damaged mitochondria for clearance via mitophagy. Impairments in fission-mitophagy coupling are well documented in AD and PD, contributing to fragmented, ROS-generating mitochondria (Wang et al., 2023; Chan, 2020; Tilokani et al., 2018). Key mitophagy pathways—including PTEN-induced putative kinase 1–Parkin (PINK1–Parkin), BCL2/adenovirus E1B 19 kDa interacting protein 3/NIP3-like protein X (BNIP3/NIX), and FUN14-domain-containing-protein-1 (FUNDC1)—prevent the accumulation of dysfunctional mitochondria. Age-related declines in these systems contribute to neurodegenerative diseases such as AD, PD, and ALS (Wang et al., 2023; Pickrell and Youle, 2015; Novak et al., 2010). Mitochondrial trafficking to dendrites and synapses is critical for local ATP supply and calcium buffering, explaining the high vulnerability of neuronal projections to energy failure and oxidative stress (Rangaraju et al., 2019). Neuronal metabolism is compartmentalized: somata rely mainly on aerobic glycolysis to limit ROS, while synapses utilize localized oxidative phosphorylation to meet rapid energy demands (Wei et al., 2023; Rangaraju et al., 2019). Electron transport chain (ETC) activity generates ATP but also produces ROS, primarily at complexes I and III (Murphy and Hartley, 2018). Antioxidant systems, including superoxide-dismutases (SOD1/2), glutathione-peroxidase (GPx), and peroxiredoxins, neutralize ROS, while physiological ROS levels mediate critical signaling through AMP-activated-protein-kinase (AMPK) and Nuclear-factor-erythroid-2–related-factor-2 (NRF2) (Holmström and Finkel, 2014; Tonelli et al., 2018). Chronic oxidative stress overwhelms these defenses, damaging lipids, proteins, and DNA (López-Otín et al., 2016). Nutrient availability modulates mitochondrial dynamics: scarcity activates AMPK, promoting fission and mitophagy, whereas nutrient abundance stimulates fusion and PGC-1α-driven biogenesis (Jornayvaz and Shulman, 2010; Misgeld and Schwarz, 2017). This coupling of structure and metabolism allows neurons to respond dynamically to energetic demands and stress, emphasizing mitochondria as key regulators of resilience and vulnerability.

### Mitochondrial alterations in CNS disorders

Mitochondrial dysfunction is a central unifying mechanism across AD, PD, HD, ALS, and multiple sclerosis (MS), manifesting as electron-transport-chain (ETC) deficits, fission/fusion imbalance, impaired mitophagy, calcium dysregulation, and oxidative stress. These defects compromise energy supply, synaptic function, and cellular survival, while mitochondrial DNA (mtDNA) copy number changes correlate with disease progression and neuronal vulnerability (Cerantonio et al., 2024; Bustamante-Barrientos et al., 2023; Meng et al., 2025). In AD, ETC deficits (especially complexes I and IV) combine with excessive fission and impaired mitophagy to produce fragmented, ROS-generating mitochondria. Dysregulated mitochondria-associated-endoplasmic-reticulum-membranes contacts (MAMs) exacerbate calcium overload and apoptosis, while mtDNA depletion worsens bioenergetic stress (D’Alessandro et al., 2025; Wang et al., 2025; Skawratananond et al., 2025). In PD, selective dopaminergic neuron loss is linked to Complex I dysfunction, *α*-synuclein-mediated transport impairments, and defective PINK1-Parkin mitophagy, while systemic mitochondrial deficits indicate broader metabolic involvement (Moradi Vastegani et al., 2023; Henrich et al., 2023). HD features mutant Huntingtin protein (mHTT)-induced oxidative phosphorylation impairment, excessive fission, defective fusion, and PGC-1α downregulation, promoting oxidative stress and apoptosis (D’Egidio et al., 2025; Joshi et al., 2025). In ALS, motor neurons exhibit ETC defects, calcium dyshomeostasis, ROS accumulation, and mislocalized proteins, including SOD1, TAR DNA-binding protein 43 (TDP-43), and Fused in Sarcoma (FUS), leading to distal energy failure and excitotoxicity (Zhong et al., 2024; Provenzano et al., 2023). In MS, axons - particularly when demyelinated - experience energetic failure, mitochondrial fragmentation, and inflammation, further exacerbating oxidative injury and neurodegeneration (Woo et al., 2024; López-Muguruza and Matute, 2023). Across these disorders, mitochondrial failure drives ATP depletion, ROS accumulation, calcium overload, and activation of apoptotic cascades. Selective vulnerability of specific neuronal populations explains disease-specific clinical phenotypes, while shared pathways highlight mitochondria as central therapeutic targets.

### Mitochondrial dysfunction in retinal degeneration

The retina, as a CNS-derived structure, exhibits high metabolic specialization and vulnerability. Photoreceptors and RPE are among the body’s most metabolically active cells, relying on dense mitochondrial populations to sustain oxidative phosphorylation for phototransduction, outer segment renewal, and visual cycle maintenance (Duarte, 2021; Giacomello et al., 2020). In particular, RPE cells play a central role in retinal metabolic homeostasis by regulating mitochondrial distribution, redox balance, and metabolic support to photoreceptors, processes that are essential for retinal integrity and function (Longoni and Demontis, 2023). This high metabolic demand comes with vulnerability. In fact, in the retina, constant light exposure, high oxygen consumption, and polyunsaturated fatty acid–rich membranes promote oxidative stress, whereas limited glycolytic buffering capacity and tightly regulated calcium handling render retinal mitochondria particularly vulnerable to metabolic and redox perturbations (Longoni and Demontis, 2023; Neto et al., 2024).

Retinal diseases illustrate how subtle mitochondrial dysfunction can have profound consequences. In AMD, RPE mitochondria are impaired by oxidative stress, endoplasmic reticulum (ER) stress, and defective autophagy, triggering feedback loops that accelerate degeneration (Qu et al., 2024; Lenin et al., 2023). DR involves hyperglycemia-induced mitochondrial fragmentation, ROS accumulation, and neurovascular compromise (Wu and Zou, 2022; Kowluru et al., 2024) In glaucoma, retinal ganglion cells rely heavily on mitochondrial ATP; Complex I deficits, ROS, mtDNA release, and impaired mitophagy drive progressive optic nerve degeneration (Yang et al., 2024; Chaphalkar et al., 2024). RP demonstrates the interplay between genetic mutation, early oxidative stress, defective mitophagy, and secondary cone degeneration, which can be mitigated by PGC-1α agonists and mitochondrial-targeted therapies (Domènech and Marfany, 2020; Hu et al., 2025). Leber hereditary optic neuropathy (LHON), caused by mtDNA point mutations, exemplifies translational mitochondrial medicine, where gene therapy and mitochondrial pharmacology partially rescue function (Battista et al., 2024; Newman et al., 2021).

The retina and CNS share susceptibility to mitochondrial dysfunction, redox imbalance, and inflammation, although the retina constitutes a highly specialized neural tissue rather than a direct model of CNS neurodegeneration, with distinct metabolic demands, cellular architecture, and stressors that shape disease mechanisms and therapeutic responsiveness. Nonetheless, those overlapping pathways might position the retina as both a model system for neurodegeneration and a tractable platform for testing interventions aimed at restoring mitochondrial resilience.

### Shared mechanistic pathways and therapeutic implications

Across CNS and retinal disorders, mitochondrial deficits—including ETC impairment, disrupted fission-fusion dynamics, defective mitophagy, calcium dysregulation, and oxidative stress—represent convergent stress pathways that lead to energy collapse, synaptic dysfunction, and apoptotic signaling (Cerantonio et al., 2024; Brandli et al., 2024; Picca et al., 2023). However, the downstream consequences and cellular vulnerability profiles of these deficits differ substantially across neuronal and glial populations. Chronic inflammation and ROS amplify this injury, while mtDNA instability reinforces selective cellular vulnerability. Recognizing these commonalities provides a framework for interventions that enhance mitochondrial quality control, antioxidant defenses, and metabolic resilience. Within this context, EE emerges as a promising approach capable of simultaneously modulating these pathways, offering a mechanistic bridge between CNS and retinal degeneration, and setting the stage for innovative, non-invasive therapeutic strategies.

## Concept of environmental enrichment (EE)

EE is an experimental paradigm originally established by Rosenzweig and Bennett in the mid-20th century, combining sensory, motor, and social stimulation to enhance brain plasticity and learning in animal models (Nithianantharajah and Hannan, 2006; Rosenzweig et al., 1978; Xu et al., 2025). EE consists of wide cages where animals are reared in large social groups and in the presence of a variety of stimulating objects that are frequently changed to ensure novelty. This environment ensures the acquisition of complex and variable information, a key aspect driving cognitive and behavioral benefits (Veissier et al., 2024). Contemporary neuroscience continues to harness EE as a powerful non-pharmacological intervention, enhancing hippocampal neurogenesis and reducing anxiety in rodents (Khalil, 2024), reinstating young cortical plasticity in adult animals (Baroncelli et al., 2010), mediating the rescue of visual functions in adult amblyopia (Sale et al., 2007), promoting favorable memory-related brain activity in older adults (Khalil, 2024), and supporting developmental and behavioral gains in infants at risk for cerebral palsy (Zhou et al., 2025) and autism spectrum disorder populations (Hernández-Arteaga et al., 2025). The therapeutic efficacy of EE emerges from the synergistic interaction of its core components: physical activity, cognitive stimulation, social interaction, and sensorial stimulation. This multi-modal approach engages a broader range of neural circuits and systemic pathways than any single component alone, making it a potent inducer of central and peripheral plasticity.

Physical activity is a foundational component of EE, often delivered via voluntary access to running wheels, tunnels, stairs, complex mazes, or varied climbing structures. In animals, physical activity increases hippocampal plasticity by enhancing BDNF expression and signaling (Vaynman et al., 2004; Ding et al., 2011). It does promote cerebral blood flow and adult hippocampal neurogenesis by stimulating neural stem cell proliferation, survival, and integration into circuitry (Gao et al., 2023). It also drives beneficial function, activating signaling pathways and downstream myokines like irisin, which enhance mitochondrial adaptation and neuroplasticity (de Oliveira Bristot et al., 2019). Endurance exercise increases hippocampal expression of Fibronectin-Type III-Domain-Containing-Protein-5 (FNDC5) (and its cleavage product irisin) under regulation; FNDC5 in turn upregulates BDNF expression, linking metabolic regulators with neurotrophic support (Wrann et al., 2013; Belviranlı and Okudan, 2018), with similar findings in Parkinson’s models following treadmill exercise (Tang et al., 2023). Exercise also improves insulin sensitivity via the AMPK/SIRT1 pathway, leading to PGC-1α activation and mitochondrial remodeling to support cognition (Vandersmissen et al., 2025). BDNF further enhances neuronal survival, synaptic plasticity, insulin sensitivity, and mitochondrial biogenesis through Tropomyosin-receptor-kinase-B (TrkB)-mediated signaling, linking metabolic changes with neural resilience (Marosi and Mattson, 2014; Toader et al., 2025).

Although individual EE components (e.g., exercise or sensory stimulation) can engage subsets of these pathways, most evidence derives from combined paradigms, limiting precise attribution of specific mechanisms to individual components.

Cognitive stimulation is achieved through exposure to novel objects, dynamic environmental configurations, and tasks that promote learning and memory, such as puzzle feeders or water mazes. These activities engage executive functions, enhance hippocampal and cortical plasticity, and reinforce synaptic connectivity. Cognitive engagement also modulates neurotransmitter systems, fosters dendritic remodeling, and increases resistance to oxidative and metabolic stress, complementing the benefits of physical exercise (Clemente-Suárez et al., 2025).

Social interaction in EE, achieved by housing animals in larger, stable groups, enhances neuroplasticity and cognitive function (Nádeníček et al., 2022). It modulates the hypothalamic–pituitary–adrenal (HPA) axis, reducing stress-induced activation and promoting a balanced neuroendocrine response (Smail et al., 2020). Social engagement also influences neuroimmune function, modulating microglial activity and cytokine signaling essential for brain health (Smith, 2021). Enriched social environments increase oxytocin and BDNF levels while reducing anxiety-like behaviors (Demirtas et al., 2025; Mora-Gallegos and Fornaguera, 2019). Moreover, it does enhance synaptic plasticity in hippocampal neural circuitries while upregulating Postsynaptic Density Protein 95 (PSD-95) and the NR2B subunit (GluN2B) of the N-methyl-D-aspartate (NMDA) receptor (Wang et al., 2020), collectively contributing to the neuroprotective profile of EE.

Sensorial stimulation provides varied input to the visual, auditory, tactile, and olfactory systems, including objects of different textures and shapes, rotating toys, auditory playbacks, and non-threatening odors. Such multisensory engagement prevents sensory deprivation, maintains the functional integrity of sensory cortices, and supports cross-modal plasticity (Lucchesi et al., 2025). In retinal degeneration, targeted visual and multi-EE may be particularly important for activating retinofugal pathways and promoting survival factor release in the visual system. Long-term exposure to visual and multi-sensory environmental enrichment in mouse models of retinal degeneration enhances retinal activity and promotes the expression of neurotrophic and survival factors, contributing to photoreceptor preservation and functional protection of the visual system (Barone et al., 2012; Dieguez et al., 2021).

EE is a holistic intervention whose whole is greater than the sum of its parts. The concurrent activation of motor, cognitive, social, and sensory pathways create a robust stimulus that engages widespread neuroplastic, metabolic, and mitochondrial adaptations, forming the foundation for its therapeutic potential across neurodegenerative and retinal disorders.

## Environmental enrichment and mitochondrial resilience: associative and mechanistic evidence

Given the multimodal nature of EE, a critical question arises: how do physical, cognitive, social, and sensory stimuli converge to produce robust neuroprotection? The answer lies in the remarkable convergence of EE’s effects on core biological pathways, generating a self-reinforcing cycle of resilience that extends from the synaptic cleft to the mitochondrial matrix. Rather than acting in isolation, EE components engage synergistically with neural support systems to orchestrate a coordinated adaptive response.

This response is fundamentally rooted in enhanced neuroplasticity. EE provides stimuli that not only increase the birth and survival of new neurons in the hippocampus but also integrate them into functional networks, supported by widespread dendritic arborization and synaptogenesis across cortical regions (Xu et al., 2025). This structural remodeling is actively sustained by a molecular *milieu* rich in neurotrophins. Upregulation of BDNF and nerve growth factor (NGF) stabilizes newly formed connections, enhances synaptic strength via long-term potentiation, and promotes neuronal survival, linking experience-dependent plasticity to cellular resilience (Neves et al., 2024).

Yet, a brain primed for plasticity is also a brain with high energetic demands. Here, EE’s profound impact on mitochondrial function becomes paramount, coupling energy requirements with the capacity to meet them. EE enhances mitochondrial efficiency by upregulating the master regulator and its downstream targets Nuclear Respiratory Factor 1 (NRF1) and Mitochondrial Transcription Factor A (TFAM), driving mitochondrial biogenesis to expand the cellular energy network and ensure adequate ATP supply for synaptic activity and neuronal maintenance (Bennett et al., 2022; Wei et al., 2024). Beyond increasing mitochondrial number, EE preserves mitochondrial quality through regulation of fission–fusion dynamics, a central determinant of neuronal health (Grel et al., 2023). Experimental studies demonstrate that long-term EE promotes a balanced state by facilitating clearance of damaged organelles through PINK1-Parkin-mediated mitophagy, while simultaneously enhancing fusion proteins such as OPA1 and MFN2 to support content mixing and resilient network formation (Zhuang et al., 2021). These processes are governed by the AMPK-SIRT1-PGC-1*α* axis, which senses energetic status and fine-tunes mitochondrial gene expression, thereby sustaining oxidative phosphorylation and the plasticity it supports (Rakshe et al., 2024).

In parallel, EE actively maintains redox homeostasis. By upregulating endogenous antioxidant defenses—including SOD2 and glutathione—EE reduces the burden of reactive oxygen species (ROS), while also enhancing both enzymatic and non-enzymatic antioxidant capacity across brain and peripheral tissues (Meng and Su, 2024; Ramos et al., 2024). This mitochondrial protection is reinforced by EE’s systemic anti-inflammatory actions, which shift microglia toward a neuroprotective phenotype and downregulate pro-inflammatory cytokines such as Interleukin IL-1β and Tumor-necrosis-factor TNF-α that impair mitochondrial function (Jiang et al., 2017; Hu et al., 2024). This establishes a virtuous cycle in which healthier mitochondria generate less oxidative stress and inflammation, further stabilizing their function.

A further, often underemphasized, consequence of mitochondrial preservation is the regulation of intrinsic apoptotic signaling. In neurodegenerative and retinal disorders, mitochondrial dysfunction promotes permeabilization of the outer mitochondrial membrane, leading to cytochrome c release and downstream caspase activation, thereby committing neurons to programmed cell death. This intrinsic apoptotic pathway is tightly controlled by the balance between pro-apoptotic factors such as Bcl-2-associated X protein (BAX) and Bcl-2 antagonist/killer (BAK) and anti-apoptotic proteins including B-cell lymphoma 2 (BCL-2) and B-cell lymphoma-extra-large (BCL-XL), and is highly sensitive to oxidative stress and bioenergetic failure (Czabotar and Garcia-Saez, 2023).

By sustaining mitochondrial membrane potential, limiting reactive oxygen species (ROS) accumulation, and preserving ATP availability, EE indirectly shifts this balance toward cell survival. In parallel, EE-driven upregulation of neurotrophic signaling—particularly BDNF-mediated activation of PI3K–AKT and ERK pathways—reinforces anti-apoptotic programs, inhibits mitochondrial pro-death signaling, and enhances neuronal resilience. Consistent with this framework, recent evidence indicates that activation of PI3K–AKT–dependent survival pathways under oxidative stress conditions promotes mitochondrial integrity and reduces apoptotic susceptibility in neural cells (Arzhanov et al., 2025). Together with improved mitochondrial quality control, these effects provide a coherent mechanistic basis by which EE attenuates mitochondria-driven apoptosis and supports long-term cellular viability.

Another critical dimension of this integrated model is stress buffering. EE modulates the hypothalamic–pituitary–adrenal (HPA) axis, attenuating excessive glucocorticoid release in response to challenge (Fan et al., 2021; Zeeni et al., 2015). This regulation is crucial, since balanced glucocorticoid signaling supports neurogenesis, synaptic plasticity, and mitochondrial integrity, whereas chronic dysregulation drives vulnerability to neurodegenerative processes.

The effects of EE are not confined to the CNS. The same pathways activated in the brain—enhanced metabolic sensing, reduced inflammation, and improved mitochondrial function—extend systemically, reflecting mechanisms of mitochondrial resilience increasingly recognized across tissues (Zong et al., 2024). These systemic adaptations foster improvements in overall metabolic health (Clemente-Suárez et al., 2024), including enhanced insulin sensitivity and more effective crosstalk between the brain, retina, and peripheral organs (Malin et al., 2022). Accordingly, EE should be viewed not as a purely neurocentric intervention but as a holistic strategy enhancing organismal resilience.

**Figure 1:**
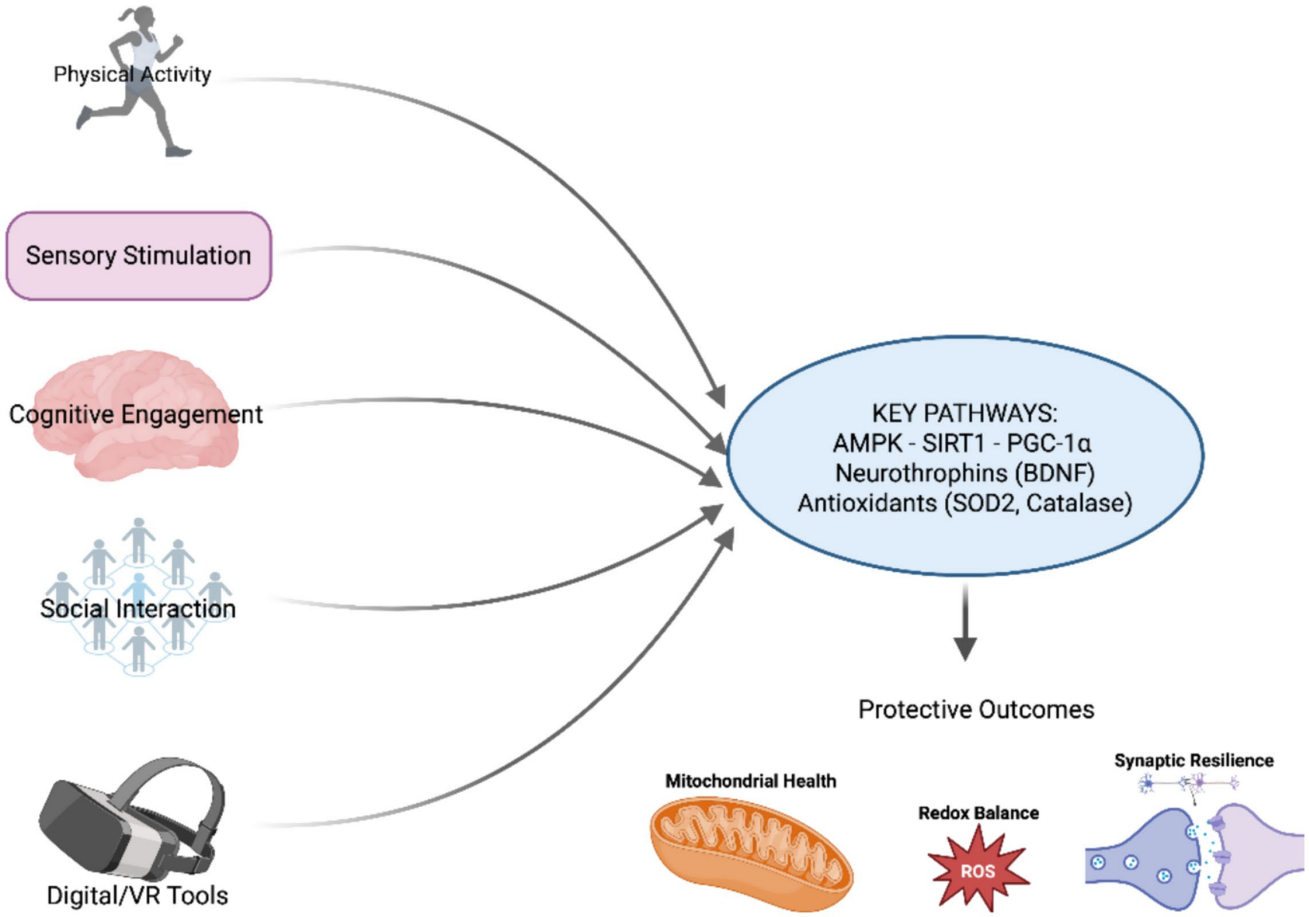
Environmental enrichment as a mitochondria-targeting systems strategy. Schematic representation of mitochondrial function under physiological conditions and mitochondrial alterations associated with neurodegenerative and retinal disorders, highlighting key nodes modulated by environmental enrichment (EE). EE is depicted as a non-pharmacological, systems-level strategy integrating physical activity, sensory stimulation, cognitive engagement, social interaction, and emerging digital/VR tools. These components converge on core molecular pathways—most prominently the AMPK–SIRT1–PGC-1α axis—regulating mitochondrial biogenesis, redox balance, quality control, and apoptotic signaling. Together, these processes support bioenergetic stability, neuronal and retinal plasticity, and cellular resilience. Direct causal links between EE and specific mitochondrial remodeling events remain under investigation: Created in BioRender. Avitabile, A. (2025) https://BioRender.com/1ju51d4.

In summary, the therapeutic potential of environmental EE lies in its capacity to simultaneously engage and synchronize the brain’s plasticity, trophic, immune, metabolic, and stress-response systems. EE does not simply combine isolated effects; it integrates them into a multilayered neurobiological framework uniquely suited to counteract the complex pathophysiology of neurodegenerative and retinal disorders. By reinforcing neuronal and mitochondrial resilience at every level, EE provides a comprehensive physiological foundation for preserving function in the face of disease. A schematic overview of normal mitochondrial function, pathological alterations, and EE-modulated pathways is provided in Figure 1.

While this scheme summarizes the principal mechanistic pathways engaged by EE, the strength, temporal requirements, and quantitative boundaries of these effects warrant explicit consideration.

In this framework, environmental enrichment is best understood not as a replacement for targeted therapies, but as a contextual modulator of biological resilience that enhances the capacity of neural and mitochondrial systems to respond to therapeutic intervention.

## Quantitative dimensions, dose–response relationships, and temporal constraints of EE

Despite the extensive body of preclinical literature supporting EE as a modulator of neural structure and function, its quantitative characterization remains limited. Most EE studies report qualitative or categorical outcomes—such as delayed symptom onset, improved behavioral performance, preservation of neuronal architecture, or enhanced synaptic plasticity—without standardized reporting of effect sizes or dose–response parameters. This methodological heterogeneity complicates cross-study comparisons and limits formal meta-analytic approaches, even within well-characterized experimental paradigms (Sale et al., 2014; Nithianantharajah and Hannan, 2006). Nevertheless, converging evidence across multiple models indicates that EE exerts reproducible, biologically meaningful effects, particularly when exposure is continuous and multimodal (Berardi et al., 2007; Spires et al., 2004; Barone et al., 2012).

Dose–response relationships in EE appear intrinsically complex and non-linear. In rodent models, paradigms combining sustained cognitive, sensory, motor, and social stimulation generally outperform intermittent or single-component interventions, suggesting that cumulative and integrated engagement is required to activate adaptive plasticity mechanisms (Sale et al., 2014; Nithianantharajah and Hannan, 2006). At the same time, preclinical evidence indicates that enrichment effects are context-dependent and constrained by the metabolic and functional reserve of the nervous system, arguing against a simplistic assumption that “more stimulation is always better.” These observations support the concept of an optimal enrichment “dose,” defined by intensity, duration, and modality composition rather than by a monotonic relationship between stimulation and benefit.

Temporal factors further shape EE efficacy. Across diverse experimental models, enrichment initiated during early or presymptomatic stages consistently produces stronger and more durable effects than interventions introduced after overt pathology has developed (Berardi et al., 2007; Spires et al., 2004; Barone et al., 2012). Once extensive neuronal loss, synaptic disconnection, or glial remodeling has occurred, enrichment-induced plasticity appears insufficient to reverse established structural damage. This temporal dependence supports the interpretation of EE primarily as a preventive or disease-modifying strategy, rather than a restorative intervention at advanced stages.

In translational contexts, quantitative assessment of EE-inspired interventions remains challenging. Human paradigms are typically time-limited, task-specific, and heterogeneous in design, with outcomes assessed predominantly through functional or behavioral measures rather than biological readouts. As a result, minimal effective exposure thresholds, dose–response relationships, and persistence of effects remain poorly defined. Disease-specific boundary conditions, negative findings, and translational limitations are discussed in detail in subsequent sections.

Collectively, these considerations highlight the need for greater quantitative rigor in future EE research. Experimental and clinical studies should explicitly define and report enrichment parameters, including duration, intensity, modality composition, and timing relative to disease onset. Longitudinal designs integrating functional outcomes with molecular and metabolic markers will be essential to establish effect sizes, therapeutic windows, and dose–response relationships, thereby advancing EE from a largely descriptive paradigm to a quantitatively grounded and mechanistically informed framework.

## Translational evidence: EE in neurodegenerative models

Environmental enrichment has progressed from a conceptual framework of sensory–motor stimulation to a translationally relevant strategy with significant disease-modifying potential across diverse neurodegenerative models. Its novelty lies not only in achieving symptomatic improvements but also in its capacity to influence core pathogenic processes, including protein aggregation, mitochondrial failure, and neuroimmune imbalance. By targeting these fundamental mechanisms, enrichment-based paradigms can interact synergistically with pharmacological and regenerative approaches, opening the door to combinatorial therapies that extend beyond what isolated interventions can achieve.

However, the transition from the laboratory to the clinic requires a nuanced understanding of how these paradigms are implemented, as rodent environmental enrichment models and human enrichment-inspired interventions are not directly equivalent. Rather, human approaches represent partial, component-specific translations of EE that engage limited subsets of the multimodal stimulation present in animal models. This distinction is vital for accurately interpreting clinical outcomes and refining future therapeutic designs.

Importantly, it must be kept in mind that the relative contribution of mitochondrial remodeling, synaptic plasticity, and immune modulation varies substantially across diseases and cannot be assumed to scale uniformly between CNS neurodegenerative disorders and retinal dystrophies.

In Alzheimer’s disease, EE extends its benefits beyond cognitive rescue to direct modulation of hallmark lesions. Enriched housing reduces amyloid-β load and tau hyperphosphorylation, changes that align with improved synaptic integrity and network function (Xu et al., 2025). Mechanistic studies reveal that EE regulates blood–brain barrier transporters such as Low-Density-Lipoprotein-Receptor-Related-Protein-1 (LRP1) and Receptor-for-Advanced-Glycation-End-products (RAGE), thereby restoring amyloid efflux and protecting against proteostatic overload (Liew et al., 2022; Xue et al., 2022). This identifies a novel mechanism, i.e., environmental modulation of proteostasis at the neurovascular interface. Evidence that EE dampens tau spreading (Stozicka et al., 2020) further suggests that it operates across both amyloid and tau axes, a rare feature for a non-pharmacological intervention.

Parkinson’s disease models similarly highlight EE’s role in stabilizing vulnerable neural circuits. In enriched housing conditions, dopaminergic networks are preserved, misfolded *α*-synuclein is reduced, and motor coordination improves alongside upregulation of protective factors such as Reelin (Cho et al., 2022). These findings position EE as a non-invasive strategy with the capacity to mitigate proteostasis stress in synucleinopathies. Huntington’s disease provides another example where the innovative dimension lies in EE’s role as a biological adjuvant. When combined with human striatal progenitor grafts, EE accelerates graft integration, promotes corticostriatal synaptic connectivity, and enhances both motor and cognitive recovery (Schellino et al., 2023). In this context, EE provides a plasticity-promoting *milieu* that maximizes the efficacy of regenerative therapies.

The influence of EE extends into demyelinating diseases. In multiple sclerosis models, enriched conditions preserve presynaptic terminals and glutamatergic transmission (Bonfiglio et al., 2019), support myelin integrity and ameliorate disease signs (Silva and Ferrari, 2020) and reduce relapse frequency and severity in relapsing–remitting paradigms through anti-inflammatory conditioning (Fournier et al., 2020). Importantly, EE has also been shown to foster remyelination, an area of high translational interest given the lack of effective remyelinating therapies (Lubetzki et al., 2020; Zveik et al., 2024).

By contrast, the evidence in amyotrophic lateral sclerosis remains less robust. EE has not consistently produced strong disease-modifying effects in ALS models, yet it engages overlapping mechanisms—including mitochondrial support, dampening of neuroinflammation, and synaptic stabilization—that suggest potential as a systems-level enhancer when integrated with targeted approaches (Vaquero-Rodríguez et al., 2023). In contrast to other neurodegenerative conditions, evidence supporting a disease-modifying role for environmental enrichment (EE) in amyotrophic lateral sclerosis (ALS) remains limited and inconsistent. In some experimental contexts, increased physical activity or stimulation has been associated with accelerated disease progression, likely due to heightened metabolic and neuromuscular stress on vulnerable motor neurons. For example, in SOD1-G93A ALS mouse models, high-intensity and endurance exercise hastened disease onset and exacerbated neuromuscular degeneration and motor neuron loss compared with sedentary conditions, suggesting that strenuous stimulation can worsen pathological features (Scaricamazza et al., 2024; Fenili et al., 2024) [e.g., high intensity exercise brought forward onset and worsened motor decline in ALS mice (Scaricamazza et al., 2024; Fenili et al., 2024)]. Moreover, reviews of physical activity as a risk factor in ALS note that strenuous physical activity is linked to increased ALS risk and may interact with genetic susceptibility, underscoring the absence of a clear protective or disease-modifying effect of high intensity EE-like stimulation in this condition (Chapman et al., 2023). These findings suggest that, in ALS, mitochondrial and energetic fragility may limit the cellular capacity to benefit from enrichment-based stimulation. Importantly, ALS pathology involves early disruption of axonal transport, calcium homeostasis, and neuromuscular junction (NMJ) integrity—processes that may not be adequately compensated by experience-dependent plasticity alone. Together, these observations highlight ALS as a boundary condition for EE efficacy and underscore the need for carefully titrated, disease-stage–specific enrichment paradigms rather than generalized stimulation.

This highlights the importance of innovative study designs that test EE not as a standalone intervention but as part of multimodal therapeutic frameworks (Masrori and Van Damme, 2020; Chiò et al., 2020). In fact, at present, it remains speculative whether these human interventions reproduce the mitochondrial, epigenetic, and systemic adaptations observed in rodent EE, as direct biomarkers of mitochondrial remodeling have rarely been assessed in clinical studies. While both may converge on shared downstream pathways—such as neurotrophin signaling or synaptic plasticity—direct comparisons of effect magnitude, duration, and systemic engagement between rodent EE and human interventions are currently lacking.

Taken together, these considerations highlight EE’s innovative conceptual edge: rather than acting as a stand-alone therapy, EE is best viewed as a system-level biological context that may influence disease trajectories and therapeutic responsiveness. By engaging pathways related to mitochondrial quality control, proteostasis, synaptic resilience, and immune tone—primarily demonstrated in preclinical models—EE may create an enabling environment in which pharmacological and genetic interventions can achieve enhanced or more durable effects. In this framework, EE is not an alternative to targeted therapies but a complementary foundation that may amplify their impact across neurodegenerative conditions.

## Emerging evidence: EE in retinal degeneration

Environmental enrichment creates a permissive biological environment that enhances the efficacy of gene- and cell-based therapies in CNS models by improving mitochondrial fitness, plasticity, and circuit integration. In retinal dystrophies, however, these potential synergistic effects remain largely hypothetical. They are currently supported by shared mechanistic principles rather than direct experimental evidence. Accordingly, the retina-related implications discussed here should be interpreted as hypothesis-generating, extrapolated primarily from CNS studies, and pending dedicated retina-specific validation.

Recent findings underscore the remarkable adaptability of the adult retina under enriched sensory conditions—an idea once considered exclusive to higher brain regions. In a striking demonstration of translational depth, adult rats exposed to enriched environments before retinal ischemia preserved electroretinogram responses and experienced reduced ganglion cell loss. These protective effects were mediated by elevated BDNF, and importantly, they disappeared when BDNF–TrkB signaling was blocked, revealing a specific molecular conduit through which sensory experience enhances retinal resilience (González Fleitas et al., 2022). Activity thus emerges not only as functional input but as a dynamic regulator of retinal health. Enriched visual and multisensory stimuli engage adaptive neurotrophic cascades that sustain retinal circuitry even under stress, reframing the retina as a plastic organ capable of experience-driven fortification.

This adaptive capacity is further highlighted by animal models of retinal disease. In diabetic retinopathy, enriched housing preserved electroretinographic function, suppressed Vascular-endothelial-growth-factor (VEGF) and TNF-*α* upregulation, reduced lipid peroxidation, and maintained BDNF levels across retinal layers, demonstrating how EE counters diabetes-induced stressors (González Fleitas et al., 2022). In glaucoma models, animals exposed to EE showed enhanced visual evoked potentials, preserved retinal ganglion cell numbers, reduced microglial reactivity, and sustained BDNF expression, pointing to a convergence of synaptic support, anti-inflammatory action, and neurotrophin-driven resilience (González Fleitas et al., 2022). Similarly, in mouse models of non-exudative age-related macular degeneration, enriched conditions protected the retinal pigment epithelium and photoreceptors through structural preservation and functional maintenance, offering one of the few demonstrations of EE directly influencing AMD-like pathology *in vivo* (Dieguez et al., 2021). Collectively, these studies illustrate that EE does not exert narrowly targeted effects, but rather orchestrates a systemic adaptive response that buffers oxidative, metabolic, and inflammatory stressors across diverse retinal insults.

However, translation of EE principles to human retinal disease has yielded mixed results. The EFFECT randomized controlled trial assessing eccentric viewing training in age-related macular degeneration failed to demonstrate significant improvements in self-reported task performance, reading speed, or fixation stability (Rubin et al., 2023). These neutral findings underscore the difficulty of translating enrichment-based interventions to advanced degenerative stages and suggest that residual neural plasticity may be insufficient to overcome structural damage. Moreover, inter-individual variability in disease severity, cognitive reserve, motivation, and adherence likely contributes to inconsistent outcomes across studies. While disappointing, these findings highlight the difficulty of counteracting progressive degeneration, though the authors suggest that such training may retain value in advanced disease stages (Rubin et al., 2023). More encouraging are studies of visual search training, where patients with central vision loss who engaged in structured exercises to improve saccadic use of their preferred retinal locus exhibited faster saccades, more directed fixations, and retained some improvements 2 to 3 months after training, even if transfer to novel tasks was limited (Janssen and Verghese, 2016). Additional layers of intervention appear to amplify such effects: transcranial random noise stimulation applied over the visual cortex during perceptual training improved contrast sensitivity in patients with bilateral macular degeneration, reinforcing the notion that multimodal strategies can enhance the perception of degraded visual inputs (Contemori et al., 2024).

Complementary non-invasive approaches have also emerged in the form of light-based mitochondrial stimulation. Photobiomodulation has shown promise in dry AMD, with a recent meta-analysis of randomized controlled trials reporting improvements in best-corrected visual acuity and reductions in drusen volume, consistent with enhanced retinal energy metabolism and structural stabilization (Rassi et al., 2024). Together, these human studies demonstrate that EE-inspired, component-based interventions can foster residual retinal–cortical plasticity, particularly when combined with metabolic or psychophysical strategies, and that they are most effective in earlier or adaptive stages of disease.

Taken together, this body of evidence illuminates how EE reshapes the conceptual landscape of retinal degeneration. EE’s strength lies in mobilizing endogenous resilience through neurotrophin induction, metabolic fortification, and immunomodulation across diverse disease contexts, including diabetic retinopathy, glaucoma, and AMD. Although human trials are at an earlier stage, emerging approaches such as perceptual remapping, neuromodulatory enhancement, and light-based metabolic support align closely with EE’s mechanistic underpinnings. Even when interventions such as eccentric viewing training yield limited results, others demonstrate that adaptive plasticity remains accessible and exploitable. Crucially, EE-inspired strategies complement rather than duplicate molecular and gene-based therapies. By fortifying the plastic substrate of surviving retinal circuits, they may enhance the durability and functional efficacy of biomedical interventions. Future directions should explore combinatorial paradigms in which EE-based rehabilitation is deliberately integrated with pharmacological, gene, cell, or biotechnological therapies catalyzing systemic resilience and redefining the therapeutic horizon in vision science.

The following section will now move beyond disease-specific retinal evidence to examine EE as a systemic and visual therapeutic strategy acting across retinal and central visual networks.

## Environmental enrichment as a systemic and visual therapeutic strategy

Building upon the experimental and disease-specific evidence in retinal degeneration models discussed in the previous section, EE has emerged as more than a laboratory curiosity; it now stands at the forefront of non-invasive and non-pharmacological interventions with systemic impact. Having reviewed the emerging experimental evidence supporting the role of environmental enrichment (EE) in retinal degeneration, this section adopts a broader, integrative perspective, positioning EE as a systemic and visual therapeutic strategy acting across retinal and central visual pathways.

Framed as a non-pharmacological “mitochondrial therapy,” EE achieves *in vivo* what drugs often seek *in vitro*—revitalizing mitochondrial biogenesis, optimizing energy metabolism, and curbing oxidative stress, all without the risks of side-effects. As detailed above in retinal degeneration models, recent rodent studies demonstrate that EE housing activates protective pathways in the retina by preventing diabetes-induced VEGF overexpression, a hallmark of early retinal compromise, and by preserving BDNF levels while diminishing inflammation and oxidative damage (Dorfman et al., 2014).

Extending the retina-specific observations summarized earlier, EE’s capacity to potentiate retinal resilience transcends diabetic retinopathy. In a model of non-exudative age-related macular degeneration (AMD), enriched environments significantly increased BDNF expression in both the retina and the RPE, providing structural and functional protection in an injury setting that mirrors early human AMD pathology (Dieguez et al., 2021). These findings embody EE’s systemic potential: by safeguarding mitochondrial and neurotrophic integrity, EE functions as a multi-system pro-resilience modality—not targeting one lesion but reinforcing defense across different degenerative axes.

## Environmental enrichment as a permissive biological context for gene- and circuit-based interventions

The potential interaction between environmental enrichment (EE) and gene-based therapies has attracted interest primarily in the context of central nervous system (CNS) disorders, where the functional integration of newly expressed or corrected genes depends not only on vector delivery but also on the metabolic and plastic state of the host tissue. Importantly, direct evidence that EE enhances the efficacy of retinal gene therapy is currently lacking, and retina-specific conclusions cannot be drawn at this stage.

In CNS models, enriched environments have been shown to improve neuronal survival, synaptic integration, and circuit-level plasticity following gene transfer or regenerative interventions. These effects are generally attributed to EE-induced improvements in mitochondrial efficiency, neurotrophic support, and activity-dependent synaptic stabilization, rather than to any direct interaction with gene delivery mechanisms themselves. EE, in this context, is best understood as a permissive biological milieu that may facilitate functional outcomes once genetic correction has occurred, rather than as a modifier of gene expression per se.

Whether similar principles apply to retinal gene therapy remains an open question. Retinal neurons exhibit distinct metabolic constraints, limited regenerative capacity, and highly specialized circuit architecture, all of which may restrict the translatability of CNS findings. Moreover, no convincing evidence currently supports a role for EE in mitochondrial optic neuropathies such as LHON, and such conditions should not be inferred to benefit from enrichment-based interventions in the absence of targeted experimental validation.

Accordingly, any proposed interaction between EE and retinal gene therapy should be regarded as conceptual and hypothesis-generating, grounded in shared mechanisms of mitochondrial resilience, synaptic activity, and neurotrophic signaling rather than in direct experimental proof. Future studies specifically designed to test whether EE can modulate post-therapeutic outcomes—such as durability of gene expression, circuit stabilization, or functional recovery—will be required before meaningful translational claims can be made.

In this framework, EE is not positioned as an enhancer of gene therapy efficacy, but rather as a contextual modifier of tissue responsiveness, whose relevance to retinal gene therapy remains to be established through retina-specific experimental and clinical studies.

These observations support a broader conceptual framework in which the biological state of the host tissue—shaped by experience-dependent plasticity—may influence therapeutic responsiveness, even in the absence of direct effects on gene delivery or expression (Xu et al., 2025).

Beyond the retina-focused experimental studies discussed above, EE also shows promise when integrated with pharmacological or gene-based therapies. EE has been shown to synergize with gene therapy, stem cell transplantation, and regenerative strategies in CNS disease models, leading to improved functional outcomes (Xu et al., 2025). Combining EE-inspired interventions with emerging retinal gene and cell therapies represents a promising but as yet unvalidated strategy. Determining whether EE can extend therapeutic durability or efficacy in retinal diseases will require dedicated, retina-specific experimental studies. However, although direct retina-specific combinatorial studies remain limited, the broader literature strongly suggests synergy. For instance, in the CNS, enriched environments have been shown to facilitate the survival and integration of grafted neural progenitors, largely by enhancing synaptic plasticity and metabolic resilience. As reviewed by Han et al. (2022), these findings underscore a broader translational principle: by creating a receptive host environment, EE may likewise render ocular tissues more amenable to gene therapy vectors or mitochondrial-targeting drugs.

Beyond biochemical resilience, EE exerts profound effects on neural connectivity. Enrichment paradigms preserve cortico-retinal communication and mirror findings from other sensory systems, where EE enhances synaptic complexity and capillary density in sensory cortices, thereby improving signal fidelity (Alwis and Rajan, 2014). More recently, vision research has been framed as a window into broader brain plasticity, underscoring how retinal and cortical circuits adapt in parallel to environmental modulation (Ayoub, 2025). Together, these findings suggest that EE fosters not only retinal protection but also the integrity of higher-order visual pathways, strengthening the bidirectional dialogue between eye and brain.

Rodents exposed to enriched environments from prenatal stages through adulthood develop enlarged primary visual cortices and broader visual field representations, indicating enhanced retinocortical mapping and structural organization (Bibollet-Bahena et al., 2023). In humans, aerobic exercise has been shown to promote visual cortex plasticity, suggesting that sensory or physical stimulation can support cortical adaptability in adults (Abuleil et al., 2022). In human settings, further disentanglement of individual EE components is complicated by variability in intervention design and participant engagement, reinforcing the need for component-specific and factorial study designs.

Although direct studies of EE’s effects on retina-to-brain connectivity in degenerative models remain limited, these findings highlight a credible mechanistic basis: by reinforcing the integrity of visual cortical circuits, EE may help preserve functional vision despite retinal stress. This connectivity-focused approach is particularly relevant for diseases like glaucoma, where the disconnection between retinal ganglion cells and central visual pathways contributes substantially to vision loss. By supporting both retinal cells and their cortical synapses, EE holds promise as a functional stabilizer and holistic therapeutic partner.

Consistent with the experimental retinal evidence reviewed earlier, voluntary exercise in mouse models suppresses laser-induced choroidal neovascularization—a surrogate for neovascular AMD—through immunomodulatory mechanisms, including reduced macrophage infiltration and lowered expression of angiogenic and inflammatory cytokines (Makin et al., 2020), and inhibition of the AIM2 inflammasome mediated by adiponectin release (Cui et al., 2023). These findings compellingly suggest that regular physical activity may intercept neovascular pathogenesis at a preclinical stage by engaging systemic anti-inflammatory pathways.

Moreover, as previously reported in retinal models, regular voluntary exercise preserves retinal thickness and electrophysiological function in aging rodents, this effect being associated with increased trophic factors, reduced oxidative stress markers, and enhanced mitochondrial biogenesis within the retina itself (Szilágyi et al., 2024). These experimental data resonate with broader reviews highlighting that physical activity promotes ocular resilience across conditions such as AMD, glaucoma, and diabetic retinopathy, acting through overlapping neurotrophic, vascular, and mitochondrial mechanisms (Zhang et al., 2024). Together, these insights position EE and allied lifestyle interventions as accessible, scalable strategies to bolster retinal resilience before overt disease onset.

Therefore, EE embodies a holistic therapeutic philosophy—one that unifies metabolic, synaptic, and connectivity-based mechanisms into a single integrated framework. As a non-pharmacological mitochondrial therapy, EE empowers retinal tissue to engage its endogenous protective machinery, modulating VEGF, BDNF, and inflammatory mediators in ways that complement—and potentially amplify—the efficacy of drugs or gene therapies. Moreover, EE’s reinforcement of retinocortical circuits reflects its unique capacity to simultaneously preserve structure and function across the visual axis. When adopted early—as preventive measure in individuals with early AMD or cognitive decline—EE-based habits may delay the tipping point into pathological degeneration. As recently summarized, exercise and EE interventions offer a versatile and mechanistically grounded avenue for ocular health promotion across diseases (Zhang et al., 2024). Thus, EE is not an alternative to biomedical therapies but a strategic ally: by enhancing the internal environment in which treatments act, it promises synergy, resilience, and long-term vision preservation.

## Pharmacological and gene therapy synergies

**Figure 2:**
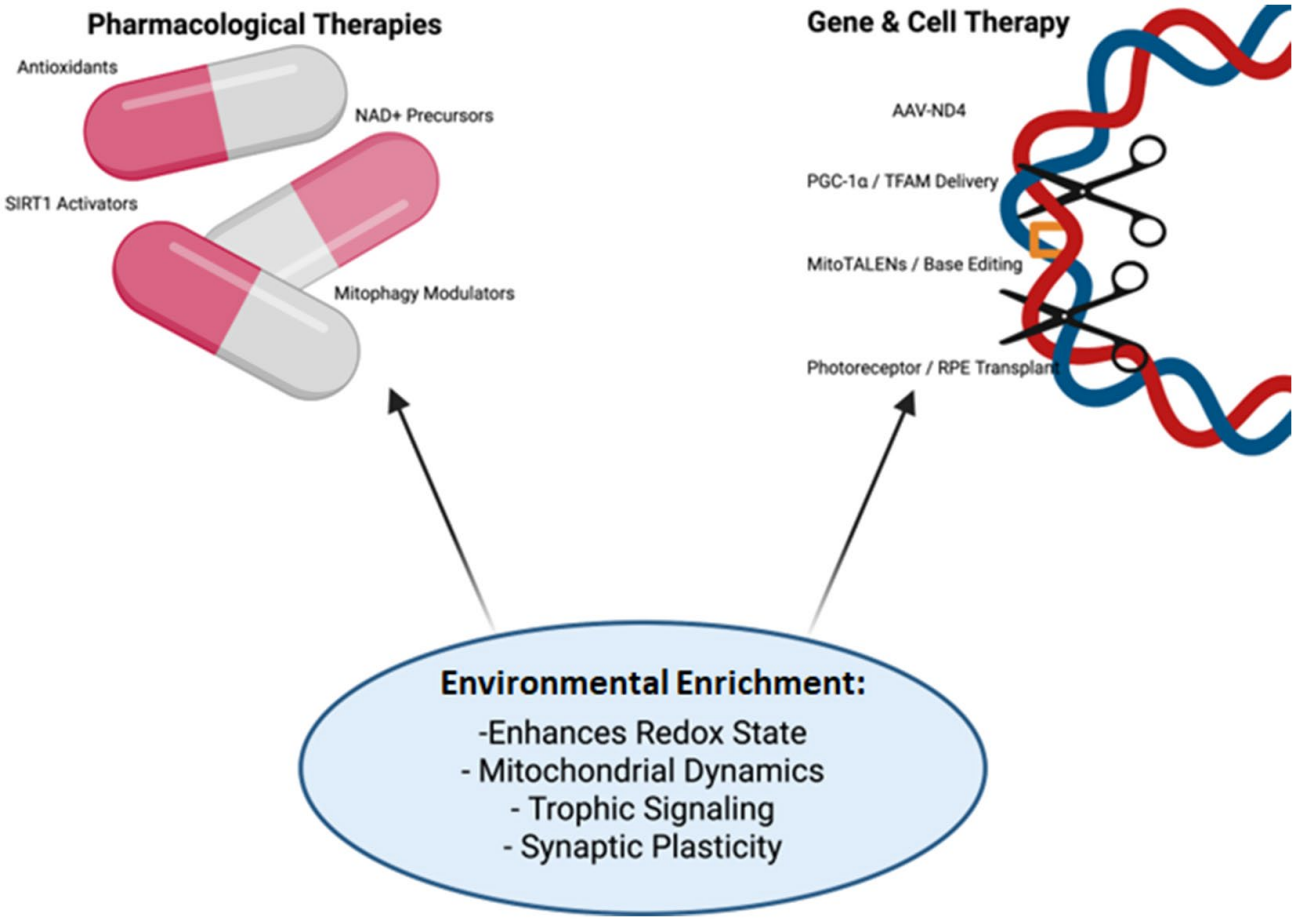
Synergistic interactions of EE with pharmacological and genetic therapies. Conceptual framework illustrating how EE functions as a biological amplifier of targeted interventions. Pharmacological therapies—including antioxidants (coenzyme Q10, MitoQ, SkQ1, NAC), NAD^+^ precursors (NR, NMN), and mitophagy modulators (urolithin A, spermidine)—operate in parallel with gene- and cell-based approaches such as AAV-mediated ND4 delivery, PGC-1α/TFAM augmentation, mitochondrial genome editing, and retinal cell transplantation. EE enhances mitochondrial dynamics, redox state, trophic signaling, and synaptic plasticity, thereby optimizing the efficacy and durability of these interventions. The diagram emphasizes the paradigm of mitochondrial combinatorial therapy, where behavioral and molecular strategies converge to reinforce therapeutic outcomes. Created in BioRender. Avitabile, A. (2025) https://BioRender.com/8oyaj9m.

The multifactorial nature of retinal degeneration makes single-target interventions insufficient. Insights from mitochondrial biology and neurodegeneration consistently show that therapeutic durability requires approaches addressing oxidative stress, bioenergetic stability, and cellular resilience in parallel (Giacomello et al., 2020; Zhang et al., 2021; Zhang et al., 2023). This perspective frames EE not as an alternative but as a systems-level adjuvant (Figure 2) that primes host tissue for greater responsiveness to pharmacological and genetic therapies (Oppici et al., 2025). Acting as a biological priming agent, EE activates endogenous resilience pathways—most prominently the AMPK–SIRT1–PGC-1α axis—that overlap with pharmacological mechanisms. By enhancing mitochondrial biogenesis, antioxidant defenses, and synaptic adaptability, it expands the therapeutic window and reduces interindividual variability in drug response (Rakshe et al., 2024; Meng and Su, 2024).

This convergence becomes particularly evident when considering mitochondria-targeting pharmacological therapies. Antioxidants such as Coenzyme Q10, MitoQ, Skulachev-ion Quinone (SkQ1), alpha-lipoic acid, and N-acetyl-cysteine (NAC) stabilize mitochondrial membranes and reduce ROS in retinal disease models (Qu et al., 2024; Kowluru et al., 2024; Brandli et al., 2024), while EE complements these effects by sustaining endogenous antioxidant systems, including SOD2 and catalase, thereby reinforcing redox stability between treatment cycles (Hu et al., 2024). Similarly, nicotinamide adenine dinucleotide (NAD^+^) precursors such as nicotinamide riboside (NR) and nicotinamide mononucleotide (NMN), along with sirtuin 1 (SIRT1) activators like resveratrol, improve mitochondrial function and DNA repair (Lee et al., 2022); EE converges on the same pathways, boosting SIRT1 activity and mitochondrial biogenesis, and thus creates a metabolic context in which these agents are more effective (Vandersmissen et al., 2025). Other compounds, including urolithin A and spermidine, support mitochondrial quality control and protect retinal cells under stress (Yang et al., 2024; Ahsan et al., 2019). Here too, EE exerts complementary effects by promoting PINK1–Parkin-mediated mitophagy and stabilizing the fusion–fission balance of mitochondria (Hu et al., 2024).

Beyond pharmacological strategies, synergies extend to gene and cell-based therapies. Clinical trials have already confirmed the efficacy of NADH Dehydrogenase Subunit 4 (ND4) gene delivery for Leber hereditary optic neuropathy (Battista et al., 2024; Newman et al., 2021), while preclinical evidence indicates that enhancing mitochondrial biogenesis and quality control through the PGC-1α–TFAM axis can reinforce cellular resilience under stress conditions, as shown by recent studies linking PGC-1α activation to improved mitochondrial metabolism and protection against degeneration (Huang et al., 2025; Chen et al., 2022). In this context, EE improves redox balance and trophic signaling, thereby providing an optimized environment for transgene expression and integration. Likewise, Clustered Regularly Interspaced Short Palindromic Repeats (CRISPR)-free mitochondrial editing platforms—including mitochondrial-targeted Transcription Activator-Like Effector Nucleases (mitoTALENs), Zinc Finger Nucleases (ZFNs), and mitochondrial base editors —have enabled precise manipulation of mtDNA in preclinical and patient-derived contexts, allowing correction of pathogenic variants and restoration of mitochondrial function (Lim, 2024; Joore et al., 2025). Although the influence of oxidative stress on editing fidelity remains underexplored, it is plausible that interventions reducing ROS and stabilizing mitochondrial dynamics—such as EE—could enhance both efficiency and durability. A similar rationale applies to cell replacement strategies: successful integration of transplanted photoreceptors, RPE cells, or retinal organoids requires trophic support and host receptivity. Evidence from Huntington’s disease models demonstrates that EE enhances graft maturation, promotes host-to-graft connectivity, and increases dendritic spine density (Schellino et al., 2023), while broader reviews indicate that EE and exercise potentiate stem-cell-based therapies across neurodegenerative conditions (Berlet et al., 2022). These findings suggest that in retinal transplantation, EE may likewise create a more receptive microenvironment for graft survival and functional integration.

At a broader regulatory level, EE also acts as an epigenetic and transcriptional enhancer (Fischer et al., 2007; Pizzorusso et al., 2007). By modulating DNA methylation, histone acetylation, and miRNA networks, EE reshapes transcriptional landscapes that reinforce neuronal resilience. Preclinical and clinical studies demonstrate that EE alters chromatin accessibility and promote functional recovery (Neves et al., 2024; Espeso-Gil et al., 2021), raising the possibility that when combined with pharmacological or genetic interventions it could improve their durability through sustained epigenetic adaptation. Timing is critical: pre-emptive or short-delay EE in retinal and brain models prolongs photoreceptor survival, increases ischemic tolerance, and prevents AMD-like damage (Barone et al., 2012; Dieguez et al., 2021; Barone et al., 2014). Such findings support the concept of “therapeutic entrainment,” in which EE acts as a metabolic preconditioner before treatment and consolidates adaptive responses when maintained during and after interventions (Picca et al., 2023).

**Figure 3:**
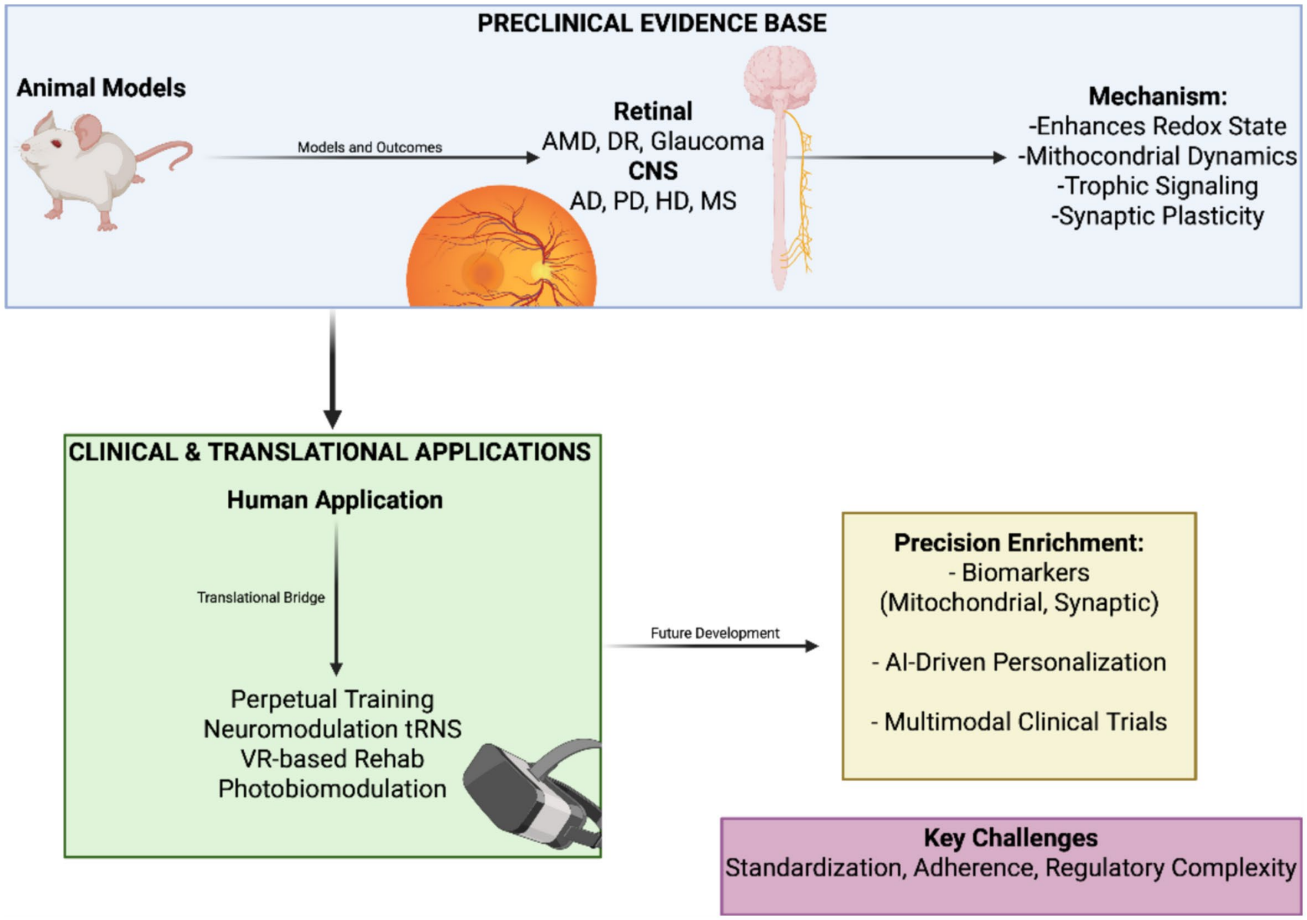
Translational trajectory of environmental enrichment: from models to clinical practice. Illustration of the translational pathway for EE in neurodegeneration and retinal disease. In preclinical models, EE protects against diverse insults, including photoreceptor loss in retinal degeneration, ganglion cell loss in glaucoma, RPE damage in AMD, protein aggregation in Alzheimer’s and Parkinson’s disease, synaptic dysfunction in Huntington’s disease, and demyelination in multiple sclerosis. In clinical and early translational settings, enrichment-inspired interventions such as perceptual training, neuromodulation (tRNS), VR-based visual rehabilitation, and photobiomodulation demonstrate feasibility and functional benefit. In future directions, EE is envisioned as part of multimodal trials integrating pharmacological, gene, and cell-based therapies, supported by biomarkers of mitochondrial and synaptic function and AI-driven personalization. The figure emphasizes EE’s role in bridging experimental discovery and precision medicine. Created in BioRender. Avitabile, A. (2025) https://BioRender.com/b2afhqs.

Translational feasibility is underscored by clinical studies (Figure 3) illustrating the feasibility of EE-inspired interventions, operating as component-based analogues of EE, in human neurodegenerative disease. Sensory-stimulation trials in Alzheimer’s disease (Chan et al., 2022) and enriched-garden programs in dementia (Bourdon and Belmin, 2021) demonstrate safety, tolerability, and preliminary functional benefits of EE-based approaches. These experiences illustrate both the promise and the logistical challenges of adherence, standardization, and regulatory complexity. As emphasized also in ALS trial design (Fenili et al., 2024), rigorous standardization will be essential for integrating EE into combination protocols. Nevertheless, the prospect of integrated neurovisual care—where behavioral and biotechnological strategies converge—represents a compelling vision for future therapeutic landscapes. EE can be regarded as a non-pharmacological “mitochondrial therapy,” but its greatest potential lies in synergy: by priming mitochondrial and epigenetic pathways, it creates a receptive cellular environment in which drugs (Maya-Vetencourt et al., 2008; Maya Vetencourt et al., 2011), nutraceuticals (Rusciano and Bagnoli, 2023; Hernández et al., 2016), genetic (Shmal et al., 2024; Campbell et al., 2016), cellular (Mead et al., 2015; Nair and Thomas, 2022), and biotechnological (Eleftheriou et al., 2017; Maya-Vetencourt et al., 2020) interventions may act more effectively. The future of retinal neuroprotection is therefore one of deliberate multimodal integration, coupling lifestyle and behavioral strategies with advanced biotechnology to forge resilience-oriented frameworks for preserving vision.

### Challenges, limitations, and future perspectives

The translation of environmental enrichment (EE) from preclinical models to clinical practice faces significant challenges. A primary barrier is the lack of standardized EE protocols. In preclinical research, EE—typically combining running wheels, tunnels, toys, and social interaction—is applied inconsistently across laboratories, complicating replication and hindering the development of scalable models tailored to specific disease stages (Oppici et al., 2025; Harland and Dalrymple-Alford, 2020).

This challenge is compounded by the translational gap between animal models and human applications. Rodents, the dominant model in EE studies, exhibit exploratory behaviors and sensory-motor adaptability that differ substantially from older human patients with retinal or cognitive disorders, limiting the predictive value of animal outcomes (Xu et al., 2025; Oppici et al., 2025). Furthermore, comprehensive rodent EE—which integrates physical activity, cognitive novelty, and social interaction in a continuous manner—is often incorrectly equated with narrower, time-limited human interventions such as perceptual training or virtual reality. This discrepancy necessitates caution when extrapolating mechanistic insights.

Available human evidence remains limited in scope, heterogeneous in design, and often indirect, with few studies reporting robust biological endpoints or long-term disease-modifying effects. Human-specific factors, including age, comorbidities, and motivational state, critically influence adherence and therapeutic efficacy (Fenili et al., 2024), further complicating quantitative evaluation. As a result, effect sizes are generally modest, outcome measures are highly task-dependent, and inter-study variability is substantial. Importantly, several trials report neutral or domain-restricted effects, underscoring the need for cautious interpretation.

Additional translational consideration concerns the baseline environmental context in which enrichment is applied. In preclinical studies, laboratory animals are typically housed in highly standardized and stimulus-poor conditions, making EE a profound deviation from baseline. In contrast, many human populations—particularly younger adults—are already exposed to high levels of sensory, cognitive, and informational stimulation in daily life, limiting the extent to which traditional EE paradigms map onto human environments. In this regard, the relevance of EE may be more pronounced in subgroups characterized by stimulus deprivation, such as elderly and socially isolated individuals, where declines in sensory, motor, and cognitive inputs coincide with age-related declines in brain function and plasticity. Aging studies show that progressive loss of sensory acuity and multi-sensory decline are associated with reduced cognitive efficacy, and that increasing environmental stimulation can aid maintenance and recovery of neural function in aged models (Leon and Woo, 2018; Wang et al., 2018).

Indeed, enriched housing ameliorates memory and social interaction deficits in aged, isolated rodents, rendering EE particularly relevant as a model for aging and sensory deprivation rather than “supraphysiological enhancement” of normal human experience (Wang et al., 2018).

From this perspective, EE-inspired strategies may operate as restorative interventions in stimulus-poor human contexts, helping to mitigate sensory and cognitive declines rather than simply augmenting already rich environments (Xu et al., 2025). Future translational efforts should therefore stratify participants by age, sensory deprivation, and baseline activity levels to better target those most likely to benefit (Yan et al., 2023). A critical limitation of the broader literature is the risk of positive reporting bias.

Building upon these translational challenges, it is evident that EE does not uniformly confer benefits. Its success hinges on a delicate interplay between disease stage, genetic background, and stimulus intensity. For instance, while cognitive engagement may be protective, excessive physical exertion can pivot from being restorative to a source of metabolic stress in a compromised system (Clemente-Suárez et al., 2024). Such findings suggest EE is not an inherently neuroprotective force but a physiological challenge requiring sufficient biological reserve for positive adaptation (Yan et al., 2023).

Consequently, the clinical application of EE-inspired paradigms often yields only modest, task-specific gains with limited generalization to overall quality of life. In progressive conditions like retinal degeneration, structural damage may outpace the potential for behavioral recovery, leading to results that lack long-term durability. These inconsistencies reinforce that enrichment operates within a narrow therapeutic window, where timing and dosage must be precisely titrated to the individual’s pathological state rather than applied as a generalized intervention (Griffin et al., 2025).

The following considerations should be understood as forward-looking and conceptual, as direct biological evidence linking AI-driven personalization or digital enrichment paradigms to specific mitochondrial or neurodegenerative outcomes remains limited.

Future progress requires addressing these limitations with greater methodological rigor. Prospective studies should incorporate objective biomarkers of mitochondrial function, synaptic plasticity, and systemic metabolism to link functional improvements to underlying biology. Digital tools, such as virtual reality (VR) and augmented reality (AR), offer promising avenues for scalable, multi-sensory stimulation, particularly for patients with limited mobility (Szczepocka et al., 2024; Gangemi et al., 2023; Yang et al., 2022). However, head-to-head comparisons are needed to determine if digital EE reproduces the molecular effects of physical paradigms, such as mitochondrial remodeling and epigenetic plasticity (Xu et al., 2025; Espeso-Gil et al., 2021). At present, most digital EE approaches demonstrate functional or behavioral adaptability rather than verified biological remodeling, highlighting the need for studies integrating molecular or metabolic endpoints.

Ultimately, integration with computational tools and artificial intelligence could predict individual adherence and biological responsiveness, enabling truly personalized, adaptive rehabilitation strategies (Costantini et al., 2024). AI-driven personalization could, in principle, help adapt EE-inspired interventions to individual behavioral and functional profiles, although direct evidence that such approaches optimize mitochondrial or neuroprotective outcomes is currently lacking. The goal is not to question whether EE exerts beneficial effects—these are well-established in models—but to operationalize it into standardized, feasible interventions. By incorporating biomarker-driven monitoring and AI-based personalization, EE may evolve from a conceptual adjunct into a practical strategy that complements biomedical innovation in neurovisual care, pending validation in biologically informed clinical studies. AI-enabled EE represents a promising conceptual framework for personalized intervention, whose biological relevance will depend on future studies linking adaptive digital environments to validated neurobiological endpoints.

These challenges are further compounded by unresolved quantitative and temporal constraints that limit the operationalization of EE in both experimental and clinical settings.

In fact, despite robust preclinical evidence supporting EE as a modulator of neural and mitochondrial resilience, its quantitative characterization remains a major translational limitation. Most EE studies rely on qualitative or categorical outcomes—such as delayed symptom onset or preserved behavioral performance—without standardized reporting of exposure intensity, duration, or effect size. This heterogeneity complicates cross-study comparison and limits formal dose–response modeling, even within otherwise well-controlled experimental paradigms.

Available evidence suggests that EE does not follow a linear “more is better” relationship. Instead, its effects appear non-linear and context-dependent, shaped by disease state, metabolic reserve, and the balance between adaptive stimulation and physiological stress. Multimodal, sustained paradigms generally outperform intermittent or single-component interventions, yet excessive or improperly timed stimulation may exceed the adaptive capacity of vulnerable systems, as illustrated by boundary conditions observed in disorders such as ALS (Clemente-Suárez et al., 2024).

Temporal factors further constrain EE efficacy. Across both CNS and retinal models, enrichment initiated at presymptomatic or early disease stages consistently produces stronger and more durable effects than interventions introduced after extensive neuronal loss or circuit disconnection (Xu et al., 2025; Oppici et al., 2025). These observations support the interpretation of EE primarily as a preventive or disease-modifying context rather than a restorative intervention at advanced stages of degeneration.

In human studies, these quantitative and temporal issues are amplified. EE-inspired interventions are typically time-limited, component-specific, and behaviorally focused, with outcomes assessed primarily at the functional level. As a result, minimal effective exposure thresholds, persistence of biological effects, and disease-specific therapeutic windows remain poorly defined (Xu et al., 2025).

Addressing these challenges will require future studies to explicitly define enrichment parameters—including duration, intensity, modality composition, and timing relative to disease onset—and to integrate functional outcomes with validated molecular, metabolic, and mitochondrial biomarkers. Such rigor will be essential to advance EE from a descriptive paradigm to a quantitatively grounded and translationally actionable framework.

## Conclusion

The evidence reviewed here highlights EE as a potent, non-pharmacological, non-invasive intervention capable of enhancing mitochondrial health and resilience across both central nervous system and retinal diseases. By engaging endogenous repair mechanisms—including neurotrophin signaling, redox homeostasis, and mitochondrial biogenesis—EE provides a systems-level therapeutic scaffold that counteracts multiple converging drivers of neuronal degeneration.

Importantly, EE does not stand in opposition to pharmacological, gene-based therapies or novel nanotechnological strategies but rather complements them. Its ability to stabilize cellular energetics, modulate immune tone, and prime epigenetic landscapes creates a biological context in which drugs, gene therapies, cell replacement strategies and nanotechnologies, can achieve greater efficacy and durability. This synergy points to the emergence of a mitochondrial combinatorial therapy paradigm, in which behavioral and molecular strategies converge to produce effects greater than the sum of their parts.

The relevance of EE is compelling in neurovisual medicine, where brain and retina share vulnerabilities rooted in mitochondrial dysfunction and chronic oxidative stress. By simultaneously targeting both compartments, EE offers a unifying approach to the prevention and treatment of degenerative disease, reinforcing the concept that vision and cognition are deeply interdependent and best supported through integrative care.

The future of vision and brain health is likely to rely on strategies that are not only curative but also preventive and personalized. Digital tools, virtual and augmented reality platforms, and AI-driven personalization offer new opportunities to develop EE-inspired, partial translational implementations of EE principles, while biomarkers of mitochondrial and synaptic function can provide objective measures of responsiveness. By combining lifestyle modulation with precision medicine, EE can be transformed from a conceptual framework into a clinically viable intervention, supporting long-term function and quality of life.

In this light, EE emerges as a foundational systems-level intervention that consistently recruits mitochondrial pathways, without yet constituting a mitochondrial therapy in the strict pharmacological sense.
